# Editorial: The role of emotional granularity in emotional regulation, mental disorders, and well-being

**DOI:** 10.3389/fpsyg.2022.1080713

**Published:** 2022-11-17

**Authors:** Yasemin Erbas, Maria Gendron, Jennifer M. B. Fugate

**Affiliations:** ^1^Tilburg School of Social and Behavioral Sciences, Tilburg University, Tilburg, Netherlands; ^2^Faculty of Psychology and Educational Sciences, KU Leuven, Leuven, Belgium; ^3^Department of Psychology, Yale University, New Haven, CT, United States; ^4^Department of Health Service Psychology, Kansas City University, Kansas City, MO, United States

**Keywords:** emotions, emotion granularity, emotion differentiation, emotion regulation, well-being, mental disorders

Imagine having a conversation with a friend. At the end of the conversation, you feel bad about it, but are not quite sure why. Now imagine having the same conversation, but afterwards you feel very guilty. In the second example, you have labeled your feelings with a high degree of specificity, and in contrast to the first example, this will aid you in dealing effectively with the situation: you might give your friend a call and apologize. This precise and context-sensitive way of labeling feelings is referred to as emotional granularity.

Two decades of research show that high emotional granularity (EG) positively relates to a wide range of well-being outcomes (Kashdan et al., 2010; Smidt and Suvak, 2015; O'Toole et al., 2020; Seah and Coifman, 2022). First, this research shows that EG is lower in individuals with various forms of psychopathology [e.g., borderline personality disorder (BPD), affective disorders, major depressive disorder]. Second, this research suggests that high EG is a protective factor, meaning that it can help to protect individuals from maladaptive behaviors or outcomes. For instance, high EG is associated with less self-harm in individuals with BPD (Zaki et al., 2013). In addition, individuals with high EG are less prone to maladaptive behaviors, such as binge eating (Dixon-Gordon et al., 2014), alcohol abuse (Kashdan et al., 2010), and physical aggression (Pond et al., 2012). Finally, there is emerging research linking EG to emotion regulation: individuals with high EG tend to report regulating their emotions more frequently (Barrett et al., 2001), while individuals with low EG are less successful in downregulating their negative emotions (Kalokerinos et al., 2019).

Together, the research on EG shows that individuals with more granular emotions have more beneficial outcomes than those individuals having undifferentiated emotions. So then why is a Research Topic on emotional granularity still necessary? One critical reason is that the general construct of emotional granularity (i.e., its nomological network) and its exact utility beyond existing ways of characterizing emotions (i.e., incremental validity) are still unclear. In addition, little is known about how EG can be cultivated, and how this could have implications for therapeutic outcomes or treatment. This Research Topic aimed to shed light on these issues, as well as to point to future directions. To summarize these aims, we have divided the papers in this Research Topic into four themes.

The first theme extends our knowledge about the relationship between high EG and improved well-being. O'Toole et al. show that EG does not predict which emotion regulation strategies at the day-level individuals use to downregulate their negative emotions. In line with earlier work at the between-person level (Kalokerinos et al., 2019), this finding suggests that the relationship between EG and well-being might have a pathway through emotion regulation that is more complex than simply the frequency or intensity of use of adaptive or maladaptive strategies. Ventura-Bort et al. provide evidence that higher granularity is related to beliefs about one's accuracy in detecting internal states—physiological and emotional—, which in turn predicted higher well-being. Furthermore, Lischetzke et al. show that higher granularity buffers against the negative effects of stress on sleep quality. Interestingly, Thompson et al. show that EG was not only low in individuals with depression, but also in those who are in remission. This finding sheds light on why individuals who have had depression in the past might be vulnerable to remission. Likewise, this finding implies that EG is thus not only a target for interventions in depressed individuals, but also in individuals who are currently in remission.

The second theme extends the benefits of having high EG to the context of therapeutic outcomes and treatment. Lazarus and Fisher find that individuals with high (compared to low) EG, especially for negative emotions, benefit more from psychotherapy. Similarly, Seah and Coifman show that individuals with Multiple Sclerosis who exhibit high EG are less likely to stop treatment when experiencing negative emotions. Together, these studies show that high EG is not only beneficial by itself, but it can also increase the likelihood to adhere to other treatment protocols.

A third theme is the malleability of EG, especially focusing on increasing low EG to affect mental well-being. Hoemann et al. show that engaging in experience sampling increases the level of EG. Likewise, Vedernikova et al. show that increasing emotion concept knowledge increases the level of negative (but not positive) granularity, with changes persisting over time. Finally, Wilson-Mendenhall and Dunne theorize how mindfulness-based interventions could cultivate EG and the specific mechanisms future research should explore.

A final theme is the continued exploration of the EG construct as it is affected by methodology, scope, and across development. Lane and Trull point out that most EG research has so far been conducted at the between-person level. However, as EG is also expected to fluctuate within individuals, they propose a new paradigm that allows to measure within-person changes in EG. Second, Nook reviews how EG develops throughout the lifespan, and the possible link to psychopathology during adolescence. Finally, a review by Tan et al. shows that positive emotion differentiation, a previously under-explored aspect of granularity, also has beneficial effects.

Together, the papers in this Research Topic showcase the different pathways through which EG can benefit emotional regulation and adherence to treatment, how EG can be cultivated, as well as identifying the existing gaps in current research with suggestions for the future. While there is consensus about the utility of EG for well-being, the pathways underlying this relationship still require study. In addition to expanding on these Research Topics, future efforts should focus on the distinction between emotional granularity and related constructs, which might help explain why retrospective survey measures about emotion vocabulary or emotional knowledge are not always strongly correlated with EG, as derived from experiential sampling methodology measures. The current Research Topic contributes to the EG literature by trying to fill parts of these gaps, but at the same time by proposing important avenues for future research.

## Author contributions

The Research Topic was proposed by JF. All editors worked collaboratively to invite potential authors and reviewers, to edit and review the manuscripts, and decide on acceptance/rejection of manuscripts. The Editorial was drafted by YE, MG, and JF. All authors contributed to the article and approved the submitted version.

## Conflict of interest

The authors declare that the research was conducted in the absence of any commercial or financial relationships that could be construed as a potential conflict of interest.

## Publisher's note

All claims expressed in this article are solely those of the authors and do not necessarily represent those of their affiliated organizations, or those of the publisher, the editors and the reviewers. Any product that may be evaluated in this article, or claim that may be made by its manufacturer, is not guaranteed or endorsed by the publisher.
